# Prediction and analysis of time series data based on granular computing

**DOI:** 10.3389/fncom.2023.1192876

**Published:** 2023-07-27

**Authors:** Yushan Yin

**Affiliations:** School of Electro-Mechanical Engineering, Xidian University, Xi’an, China

**Keywords:** granular computing, time series, large samples, machine learning, support vector machines

## Abstract

The advent of the Big Data era and the rapid development of the Internet of Things have led to a dramatic increase in the amount of data from various time series. How to classify, correlation rule mining and prediction of these large-sample time series data has a crucial role. However, due to the characteristics of high dimensionality, large data volume and transmission lag of sensor data, large sample time series data are affected by multiple factors and have complex characteristics such as multi-scale, non-linearity and burstiness. Traditional time series prediction methods are no longer applicable to the study of large sample time series data. Granular computing has unique advantages in dealing with continuous and complex data, and can compensate for the limitations of traditional support vector machines in dealing with large sample data. Therefore, this paper proposes to combine granular computing theory with support vector machines to achieve large-sample time series data prediction. Firstly, the definition of time series is analyzed, and the basic principles of traditional time series forecasting methods and granular computing are investigated. Secondly, in terms of predicting the trend of data changes, it is proposed to apply the fuzzy granulation algorithm to first convert the sample data into coarser granules. Then, it is combined with a support vector machine to predict the range of change of continuous time series data over a period of time. The results of the simulation experiments show that the proposed model is able to make accurate predictions of the range of data changes in future time periods. Compared with other prediction models, the proposed model reduces the complexity of the samples and improves the prediction accuracy.

## 1. Introduction

With the rapid development of the Internet of Things and wearable devices, more and more health data can be obtained from electronic medical records or wearable devices. Data such as heartbeat, pulse and body position changes are continuously monitored by smart wearable devices. In healthcare, much of the healthcare data is in the form of time series, such as continuous monitoring data on blood glucose, blood pressure and lipids associated with chronic diseases (Adamu et al., 2020; Das et al., 2020; Pakhchanian et al., 2021; Uslu and Stausberg, 2021). The large-sample time series data collected by wearable sensors implies a lot of very valuable human health-related information. The classification, association rule mining and prediction of these large samples of time series data are of crucial importance to the medical field (Brundin-Mather et al., 2018; Yang et al., 2018; Zhang et al., 2020).

**FIGURE 1:**
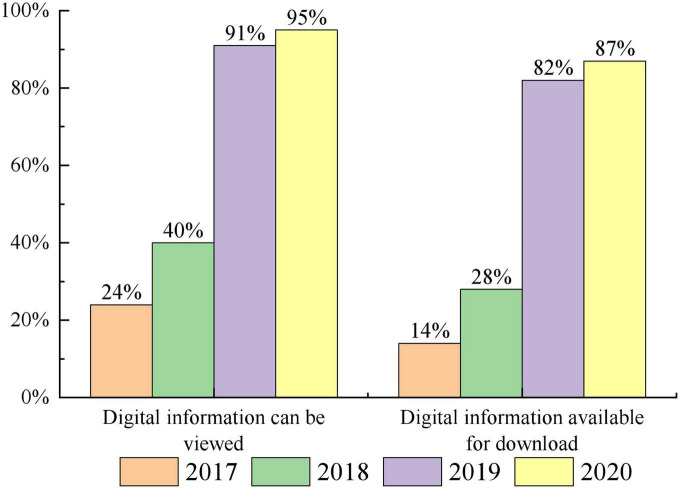
Hospitals that can provide digital healthcare information to patients.

In the medical field, there are a wide variety of sources of healthcare data. Based on the above these health care data to carry out various types of predictive model research is the current research hotspot in the field of medical information data mining (Muthee et al., 2018; Ji et al., 2019; Zhang et al., 2019; Enaizan et al., 2020). For example, according to the trend of health insurance consumption changes, the distribution of health care coverage of people in various stages of life can be analyzed to provide a theoretical basis for controlling the increase of health care costs. The use of healthcare data can predict the probability of an individual’s risk of illness, or the probability of re-admission after a patient is discharged from hospital. Through scientific and efficient analysis and mining of historical medical and health data, it can not only provide auxiliary support for early diagnosis and prevention of diseases, but also significantly reduce medical costs, thus improving the quality and efficiency of public healthcare (Chiu et al., 2018; Das et al., 2019; Ng et al., 2019; Liu et al., 2021). At this stage, more and more hospitals are providing digital healthcare information to patients, as shown in Figure 1. Accurate prediction of health time series data in real-world scenarios can provide more accurate and efficient services to a wide range of patients. Uncovering potential trends in health time series data in advance is more conducive to identifying potential disease risks at an early stage and intervening in their health (Sharp et al., 2018; Ye et al., 2019; Zhou et al., 2021).

However, due to the characteristics of wearable sensor data with high dimensionality, large data volume and transmission lag, large-sample time series data are affected by multiple factors and have complex characteristics such as multi-scale, non-linearity and burstiness. Traditional time series prediction methods are no longer suitable for the study of large-sample time series data (Fu, 2011; Gómez et al., 2016; Shrestha and Bhatta, 2018; Wauchope et al., 2021). Granular computing (Yao et al., 2013) has unique advantages in dealing with continuous, complex data and can compensate for the limitations of traditional Support Vector Machines (SVMs) (Huang et al., 2018) in dealing with large sample data. Therefore, this paper proposes to combine granular computing theory with SVM to achieve large-sample time series data prediction.

## 2. Related work

Granular computing has distinct advantages when dealing with multiple sources, heterogeneous and massive amounts of data. Granular computing can be used to granulate complex problems into a number of simple problems using the idea of “granularity” (Fujita et al., 2018; Yao, 2018; Yang et al., 2019; Liu et al., 2020). First, the computation is performed at a coarse level of granularity. Then, problems of different levels and granularity are integrated to arrive at an optimal approximate solution to the complex problem. As a result, numerous researchers have tried to introduce granular computing theory into the field of big data mining and have conducted intensive research in this direction.

In the study of data mining theory based on granular computing, Tripathy and Acharjya (2011) proposed to fuse granular computing theory with association rules, thus opening up new ideas for the application of granular computing theory in practice. Yang et al. (2020) used granular computation theory to solve the maximum flow problem in networks, which can be solved quickly and efficiently in large-scale, complex networks. Hassan (2018) combined granular computing theory with cellular automaton to effectively improve the efficiency of big data mining. The above research status shows that the theory of granular computing has made great breakthroughs and developments in the field of data mining in recent years, both in terms of theoretical research and practical applications. Granular computing models and related algorithms have also been continuously improved. How to use theory to solve practical problems is still the mainstream direction of the future development of granular computing.

Commonly used forecasting methods for time series data currently include statistical-based forecasting methods, knowledge discovery-based forecasting methods and combinatorial model-based forecasting methods. A comparison of the advantages and disadvantages of common forecasting models is shown in Table 1.

**TABLE 1:** Comparison of the advantages and disadvantages of common forecasting models.

Predictive models	Typical algorithms	Disadvantages	Advantages
Statistical models	Moving Average (MA), Auto-Regressive (AR), Auto-Regressive Moving Average (ARMA)	Only for forecasting relatively stationary data	Easy to implement and fast to achieve
Knowledge discovery model	Support vector machines (SVM), gray theory, neural networks	Difficult to solve large-scale sample classification problems	High accuracy, non-linear recognition capability
Combination models		Model selection is more difficult	Outstanding overall competence

As can be seen, typical forecasting models all have advantages and disadvantages. No model can achieve a completely idealized prediction result. Statistical models are simpler and easier to implement, but it is difficult to achieve high predictive accuracy. In addition, the generalizability of the statistical model is not high. Neural network models can have good prediction accuracy, but require a large number of samples to support them and the convergence rate is not ideal. The Auto-Regressive Moving Average (ARMA) (Hossain et al., 2020) does not require a large number of samples and can make real-time predictions and corrections to the prediction results. However, ARMA has high data requirements (Gaussian, linear). SVM is known as one of the most commonly used and effective classifiers with global optimality. SVM can successfully overcome the disadvantage that neural networks tend to fall into local minima. SVM can easily find optimal solutions on small sample training sets and has excellent generalization ability. Chen et al. (2005) proposes a fuzzy system called fuzzy support vector machine (FSVM) to deal with the unreliable generalization ability of SVMs when selected randomly to classify data examples. Margin values from three different SVMs are fuzzified, combining with the accuracy information of each SVM. The final decision is determined based on all of the SVMs. Experimental results show that the proposed fuzzy SVMs are more stable and reliable than randomly selected SVMs.

Granular computing can be divided into non-fuzzy granulation and fuzzy granulation. In the actual situation, fuzzy granulation cannot fully reflect the characteristics of things, so for most studies lacking prior information, fuzzy granulation is closer to reality than fuzzy granulation. Fuzzy granulation has the unique ability of data compression interval, and often forms a combined prediction model with SVM algorithm, which is widely used in wind speed forecasting, load forecasting, stock price forecasting and urban traffic flow forecasting. For example, Ruan et al. (2013) proposed a fuzzy granular support vector machine (FGSVM) to predict large-scale and nonlinear time series with noisy data. Compared with ordinary SVM, FGSVM has faster prediction speed. Ding et al. (2015) provides a literature survey of the hybrid models of granular computing and support vector machine. It briefly introduces three typical granular computing models and the basic theory of SVM. Then, it reviews the latest progress of these hybrid models, including fuzzy SVM, rough SVM, quotient space SVM, rough fuzzy SVM, and fuzzy rough SVM.

In order to obtain higher prediction accuracy and at the same time avoid a large number of calculations during the processing of large sample time series data, this paper proposes to combine the Fuzzy set model (Lin et al., 2021) and SVM to improve the efficiency of processing large sample time series data. The idea of fuzzy granulation of fuzzy sets can convert large-scale data into coarser individuals. Solving the problem at coarse granularity can solve the problem of low efficiency of SVM in dealing with large-scale data. After validation by simulation experiments, the proposed combination model obtains higher accuracy and prediction precision.

The main innovations and contributions of this paper include.

(1) The current research status of the mainstream models used for time series data forecasting is analyzed, and the advantages and disadvantages of the mainstream forecasting models are analyzed. The innovative new idea of combining granular computing with SVM for forecasting large sample time series data is proposed.

(2) A prediction model based on fuzzy granulation and SVM was developed. By fuzzy granulation of the sample data, the data of a window is granulated into a fuzzy interval and SVM is applied to predict the trend of data change in the future time.

## 3. Traditional time series forecasting methods

Forecasting as a science was born in relation to weather forecasting. With the advent of techniques such as meteorology, mathematical statistics and machine learning, the research direction of weather forecasting has been broadened, enabling a new level of forecast accuracy to be stepped up. A large number of real-life forecasting problems are similar to weather forecasting. The data all contain a time component, making such time-series forecasting problems even more complex. It has become a major research direction in the field of data mining to explore the potential patterns in time series data more precisely.

### 3.1. Time series definition

Time series data are a series of data values indexed in time order (Shuai et al., 2017; Epskamp et al., 2018). The most common case is observations sampled at successively equally spaced time points. Time series are generally uncertain because they are subject to intrinsic or extrinsic factors when sampled. However, there are some potential specific relationships between data at similar points in time. By exploring potential patterns in the time series through different analysis and prediction methods, future trends in the system can be predicted. Based on the potential patterns uncovered valuable predictions can be made about future trends in time series data.

For some observed variable *y*(*t*), the data recorded at different time points *t* is *y*(*t*) (*t* = 1,2,…,*n*). We call this set of data a discrete time series. Before the raw time series data is analyzed, the stationarity of the time series data is checked. In general, we consider a sampled series of a variable to be stationary if the system parameters and external conditions do not change. However, this is only a qualitative analysis and some statistical characteristics of the time series need to be tested.

The joint distribution of a strictly stationary time series is invariant under different time shifts. For a strictly stationary time series, there is no change in trend. The relationship between the mean, variance and serial continuous terms of {Y} are invariant. Due to the stringent conditions for strict stationaryness, the majority of time series in practical scenarios are not strictly stationary. In practical scenarios we commonly use weakly stationary time series (Cook et al., 2019).

The expectation, variance and covariance of the weakly stationary time series {Y} do not change over time.


(1) $$E\,\left(y_{t}\right)=\mu ,V\,a\,r\,\left(y_{t}\right)=\sigma^{2},C\,o\,v\,\left(y_{t},y_{s}\right)=f\,\left(t-s\right)$$

If the time series {Y} satisfies the above conditions, it is said to be a weakly stationary time series (a broadly stationary time series).

If a time series passes the stationaryness check, it can be modeled and predicted by a classical fitting model. However, for unsteady time series, the original time series needs to be pre-processed to convert the time series into a steady time series and then re-modeled.

### 3.2. Time series prediction based on machine learning

A stationary time series can be considered as a form of statistical equilibrium. Statistical properties such as the mean and variance of a stationary time series are not time dependent. Stationaryness is also a prerequisite for the construction of time series forecasting models. In addition, the use of stationary time series can reduce the complexity of fitting models.

The models commonly used in time series forecasting are Moving Average (MA), Auto-Regressive (AR) models, Auto-Regressive Moving Average (ARMA) models, and Auto-Regressive Integrated Moving Average (ARIMA) models. The predicted value in an AR model is made up of a linear combination of *p* observations, random errors, and a constant.


(2) $$y_{t}=c+\varphi_{1}\,y_{t-1}+\varphi_{2}\,y_{t-2}+\ldots \,\varphi_{p}\,y_{t-p}+\varepsilon_{t}$$

where *y*_*t*_ and ε_*t*_ are the predicted value and random error at time *t*, respectively, φ_*i*_ is the autoregressive coefficient and c is a constant term. However, the constant term is usually omitted in practice for the sake of simplicity. To estimate the parameters of an AR model for a given time series, the Yulc-Walker equation is usually used.

The MA model uses a set of recent observations to predict the value at a subsequent point in time. The efficient integration of AR and MA models can result in a general class of efficient time series forecasting models, called ARMA.


(3) $$y_{t}=c+\varepsilon_{t}+\sum_{i=1}^{p}\varphi_{i}\,y_{t-i}+\sum_{j=1}^{q}\theta_{j}\,\varepsilon_{t-j}$$

**FIGURE 2:**
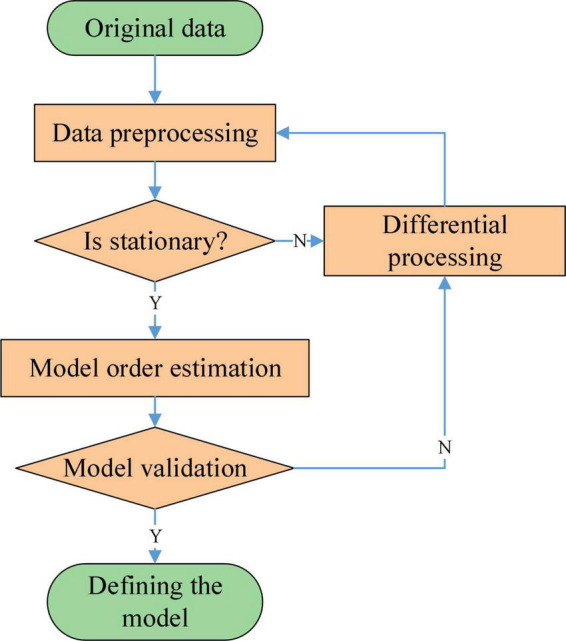
Flow of the ARIMA model.

A non-stationary time series can be converted into a stationary time series after performing multiple difference operations on it. The flow of the ARIMA model is shown in Figure 2.

The data set needs to be pre-processed with the necessary filtering and cleaning before the experiment begins. At the same time, in order to improve the efficiency of the operation, the time series need to be zero-mean processed. After zero-mean processing, the range of values of the data will be reduced, but the original pattern of variation of the data will not be changed. For non-stationary time series, it is necessary to consider the periodic variation or introduce the difference operation when pre-processing in order to reduce the impact of non-stationary on the time series. For non-stationary time series data, a stationary time series data is obtained by differencing operations. Commonly used tests for stationaryness are the time series plot test, the characteristic root test and the unit root test.

At present, in addition to statistical methods such as ARIMA, exponential moving average model and Bayesian nonparametric model, other prediction methods based on machine learning have been put forward and tested in various fields, such as neural network, deep learning, LSTM, reinforcement learning and fuzzy system. Gallos et al. (2021) presents a study on constructing functional connectivity networks (FCN) from resting-state functional magnetic resonance imaging (rsfMRI) data using manifold learning algorithms. Papaioannou et al. (2022) proposes a three-tier numerical framework based on nonlinear manifold learning for the forecasting of high-dimensional time series. The framework involves embedding the high-dimensional time series into a reduced low-dimensional space using nonlinear manifold learning, constructing reduced-order surrogate models on the manifold, and solving the pre-image problem to lift the embedded time series back to the original high-dimensional space using radial basis function interpolation and geometric harmonics.

### 3.3. Fundamentals of granule calculations

Currently, granular computing is a hot topic of research in the field of artificial intelligence and is widely used in the solution of complex problems. Starting from a practical problem, granular computing can decompose a complex problem into a simple sub-problem and replace the optimal solution with a satisfactory one. Granular computing is a new way to simulate human thinking and a powerful tool to deal with massive, fuzzy, uncertain and incomplete data. The main models in the theoretical system of granular computing are: rough sets, fuzzy sets, quotient spaces, etc.

Granular calculations consist of three main components: the granule, the granule layer and the granule structure. The granule is the most initial concept in the granule computing model and the most fundamental unit in the solution of complex problems. There are coarse and fine distinctions between granules. A granule layer is an abstract way of describing a complex problem space. It is clear from the concept of a granule layer that it is still not a structurally uniform whole. Therefore, the concept of grain structure is derived from the concept of grain layer. A granule structure is a relational structure consisting of interconnections between granule layers. It describes the structural relationships between the layers. The more complex the granule structure, the more complex the problem solving process will be. Therefore, it is important to maintain a high degree of independence and low coupling between granules at the same level in order to simplify the solution process.

The first problem to be solved in granular computing is granulation. In simple terms, the problem of granulation focuses on the selection of a suitable granulation criterion for the construction of information granules. The most basic requirement for a granulation criterion is that the granulated object must be able to fully characterize the original data. Granule calculation is generally based on a bottom-up approach. Generally, the solving process is to divide a specific problem into several granules and solve each granule. The solution for the corresponding granule layer is then synthesized according to the corresponding criterion. Finally, the final solution of the entire solution space is synthesized from the solutions of the individual grain layers. Thus granular computing can be used to solve complex problems scientifically. Currently, there are a number of widely used granulation methods: relational granules based on equivalence relations, neighborhood granules based on neighborhood systems and fuzzy information granules based on fuzzy sets.

### 3.4. Fundamentals of fuzzy sets

In the natural sciences and in the study of practical problems, the phenomenon of “fuzziness” can be found everywhere. Fuzziness refers to the fact that the degree of difference between objects of study cannot be described by the exact mathematical theory of classical sets. In order to deal with these ‘fuzzy’ phenomena, the theory of fuzzy sets has been developed, which can be used in many areas of machine learning to give rational decisions under imprecise circumstances. A fuzzy set is a collection of fuzzy concepts that can be used to express fuzziness.


(4) $$A=\left\{x\,\mu_{A}\,\left(x\right)\right\},\forall x\in X$$

where *X* is a finite non-empty region, *A* is a fuzzy set on *X*, and μ_*A*_(*x*) is the membership grade of *A*.

If μ_*A*_(*x*) = 1, then *x* is considered to belong to *A* completely. If μ_*A*_(*x*) = 0, then *x* is considered not to belong to *A at* all. If 0 < μ_*A*_(*x*) < 1, then *x is* considered to belong to *A* to some extent (μ_*A*_(*x*)). From the definition of a fuzzy set, it can be seen that the difference between an ordinary set and a fuzzy set is the range of values of the characteristic function. The former is the set {0,1} and the latter is the closed interval [0,1].

The fuzzy set *A* has different representations in different contexts, the three most commonly used representations being.

(1) Zadeh representation.


(5) $$A=\frac{A\,\left(x_{1}\right)}{x_{1}}+\frac{A\,\left(x_{2}\right)}{x_{2}}+\ldots +\frac{A\,\left(x_{n}\right)}{x_{n}}$$

Where, *A* (*x*_*i*_) is the membership function of the fuzzy set *A*.

(2) Sequential couple representation.


(6) $$A=\left\{x_{1},A\,\left(x_{1}\right),x_{2},A\,\left(x_{2}\right),\ldots ,x_{n},A\,\left(x_{n}\right)\right\}$$

(3) Vector representation.


(7) $$A=\left(A\,\left(x_{1}\right),A\,\left(x_{2}\right),\ldots \,A\,\left(x_{n}\right)\right)$$

## 4. Fuzzy granulation and SVM based prediction of large sample time series data

### 4.1. Support vector regression

Support vector machines (SVMs) are generalized linear classifiers suitable for binary classification situations. The basic principle of SVM is to find an optimal classification hyperplane that satisfies the classification requirements. The hyperplane maximizes the blank area on both sides of the hyperplane while maintaining classification accuracy. The optimal hyperplane is the most fault-tolerant to local perturbations of the training samples and produces the most robust classification results.

**FIGURE 3:**
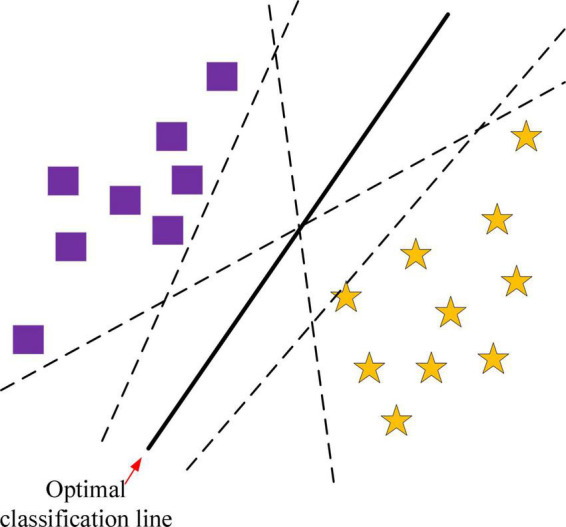
Schematic diagram of binary classification principle based on SVM.

Two classification based on SVM is shown in Figure 3. The squares and stars represent the two types of training samples, respectively. The line that can completely separate the two types of samples is called the classification line. But the optimal classification line not only separates the samples exactly and without errors, but also classifies the samples with the maximum interval between them. It can be seen that there can be an infinite number of classification lines, but only one optimal classification line. If the classification line is applied to a higher dimensional space, the classification line becomes a classification surface.

Support vector regression (SVR), developed from SVM, has good fitting power and is superior for regression estimation, time series prediction problems, etc. The basic principle of SVR is to map data to a higher dimensional feature space through a non-linear mapping. the goal of SVR is to find the optimal function from a set of function spaces.


(8) $$f\,\left(x\right)=w^{T}\,X+b$$

where ***X*** denotes the training set, ***w*** denotes the weight vector and b denotes the bias.


(9) $$\frac{1}{2}\,w^{2}+C\,\sum_{\text{i}=1}^{n}L\,\left(y_{i},f\,\left(X_{i}\right)\right)$$

where *f* denotes the optimality function, C denotes the equilibrium factor and L() denotes the loss function.

The kernel function in SVM is to map the original linear inseparable sample features into a high-dimensional space, so that it becomes linear separable in high-dimensional space. Kernel function is a function used to express the similarity between two samples, which can calculate the inner product of samples in high-dimensional space, thus avoiding the process of directly calculating high-dimensional space and greatly improving the efficiency of the algorithm. The use of kernel functions allows the SVR to map non-linear relations to higher dimensional spaces.


(10) $$f\,\left(x\right)=\sum_{i=1}^{n}\left(\alpha_{i}-\alpha_{i}^{*}\right)\,k\,\left(x_{i},z\right)+b$$

Both α_*i*_ and $\alpha_{i}^{*}$ denote Lagrange multipliers and *k*(*x*_*i*_, *z*) denotes kernel functions. Since radial basis kernel functions (also known as Gaussian kernel functions) have been widely used in time series prediction, radial basis kernel functions are also used in this paper.


(11) $$k\,\left(x_{i},x_{j}\right)=\exp\left\{-\frac{{\left|x_{i}-x_{j}\right|}^{2}}{2\,\sigma^{2}}\right\}$$

where σ denotes the radial base radius. The SVR can produce a unique global minimum solution.

This study mainly focuses on the large sample time series data from medical monitoring cases, and the corresponding constrained optimization objective function is as follows:


(12) $$\begin{array}{c} \text{Minimize}\,\psi \,\left(w,b,\xi_{1},\mu_{1}\right)=\frac{1}{2}\,w^{T}\,w+C\,\sum_{t=1}^{N}\mu_{t}\,\xi_{t}\,\\ \mathrm{Subject}\,\mathrm{to}\,\xi_{i}\geq 0\,i=1,\ldots ,N\,y_{i}\,\left(w^{T}\,\varphi \,\left(x_{i}\right)+b\right)\geq 1-\xi_{i} \end{array}$$

The objective function consists of two terms: the first term is a regularization term that penalizes large values of the weight vector *w*, and the second term is a loss term that penalizes misclassifications. The parameter *C* controls the trade-off between the two terms. The constraints in the optimization problem ensure that the hyperplane separates the data points correctly. Non-negative parameter *ξ*_*i*_ is the slack variables.

### 4.2. Information granulation algorithm based on fuzzy sets

Support Vector Machines can easily find optimal solutions on small sample training sets and have excellent generalization capabilities. However, SVM’s advantages are not as obvious when dealing with large-scale data. When dealing with large scale data, the performance of SVM may be outperformed by other models. In other words, the SVM time series forecasting model is no longer suitable for large sample time series data. Since Granular Computing has a unique advantage in dealing with continuous, complex classes of data, and can compensate for the limitations of traditional SVM in dealing with large sample data. Therefore, this paper proposes to combine Granular Computing theory with SVM to achieve large sample time series data forecasting.

Fuzzy granulation is an information processing method derived from fuzzy set theory. a method for describing information granulation was proposed by Lotfi A. Zadeh in 1979.


(13) p≜(x⁢i⁢s⁢P)⁢i⁢s⁢λ

Where *x* is a variable, *P* is a fuzzy subset, and λ is the probability that an event may occur. In most cases, the range of values of the variable is a set of real numbers. *P* is a convex fuzzy subset within the range of values, and λ is a fuzzy subset within the unit interval.

There are three main ways of using fuzzy granulation algorithms to describe information granules: time-based algorithms, numerical axis-based algorithms and combinatorial axis-based algorithms. The time-axis based fuzzy granulation algorithm used in this study.

First, the time series of the initial sample is divided into a number of small and consecutive time intervals as required by the actual problem. Each time series is the concept of a window in the conventional sense of the fuzzy granulation algorithm. The fuzzy granulation algorithm is then used to granulate the data from each window to produce a number of fuzzy information granules. The key to the fuzzy granulation process is the sample data fuzzification process. Assume a time series is *X* = (*x*_1_, *x*_2_ …*x*_*n*_). The essence of the fuzzification process is to find the fuzzy granule *G* on the time series *X*. The main fuzzy granules *G* widely used at present are triangular, trapezoidal and asymmetric Gaussian shapes. In this paper, triangular fuzzy granules are employed.


(14) $$A\,\left(x,a,m,b\right)=\left\{ \begin{array}{cc} 0, & x<a\\ \frac{x-a}{m-a}, & a\leq x\leq m\\ \frac{b-x}{b-m}, & m<x\leq b\\ 0, & x>b \end{array}\right.$$

where *A* denotes the membership function.

*G* is a subset of fuzzy information in the domain *U*. Therefore, the definition of the fuzzy particle *G* is as follows:


(15) G⁢(a,m,b)=A⁢(x),x∈X

The steps of the fuzzy information granulation algorithm are as follows.

Input: information on data based on time series.

Output: fuzzy information granules.

Step l: Solve for the parameter *m* of the fuzzy granules. rearrange the input time series in ascending time order. Assume that the rearranged order is *X* = (*x*_1_, *x*_2_ …*x*_*n*_);

Step 2: Solving for the fuzzy granule parameter *a*.


(16) $$\max Q\,\left(a\right)=\frac{\sum_{x_{k}\leq m}G\,\left(x_{k}\right)}{m-a}$$

Step 3: Solving for the fuzzy granule parameter *b*.


(17) $$\max Q\,\left(b\right)=\frac{\sum_{x_{k}>m}G\,\left(x_{k}\right)}{b-m}$$

Step 4: Get the fuzzy granule *G*(*a*,*m*,*b*);

Step 5: End of the algorithm.

### 4.3. SVM prediction model based on information granulation

Granular computing is a new direction emerging in the field of artificial intelligence. The theory of granular computing proposes new concepts and computational paradigms. In this paper, we combine the idea of granular computing with SVM to reduce the complexity of problem solving, thus effectively improving the training efficiency of SVM as well as the prediction accuracy.

**FIGURE 4:**
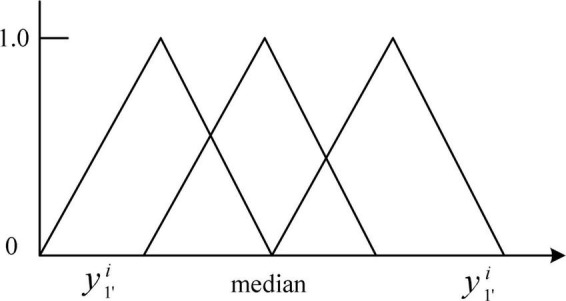
The rules of fuzzy granulation methods.

In the FGSVM hybrid model, in order to improve the prediction efficiency, the large time series is refined into sub-series. However, this method does not improve the prediction accuracy. Different from the FGSVM hybrid model, the fuzzy theory is improved in order to obtain better granulation effect. This process can be divided into two parts: The first part is to divide the original data according to certain rules and determine the best time window size. The second part is to determine the information granulation rules suitable for the original data, and the best membership function can ensure the superiority of data granulation. Building a fuzzy information granulation model based on time series data can be divided into two steps: Determine the time window partition and build membership function. The fuzzy granulation methods for the values is shown in Figure 4.

**FIGURE 5:**
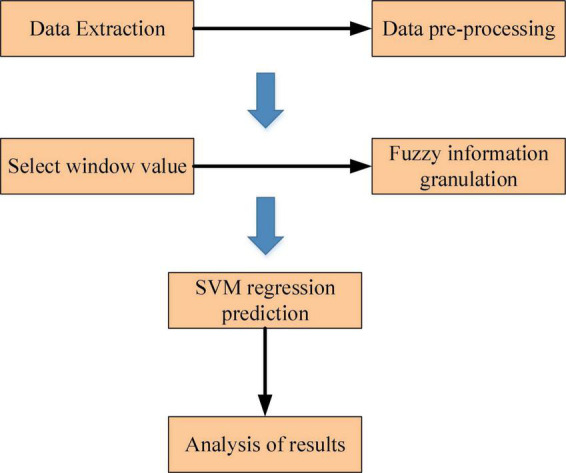
Flow of the proposed GC-SVM combination prediction model.

For large sample time series data, the process of the SVM prediction model based on information granulation is divided into two steps. The first step is to apply the fuzzy set-based information granulation algorithm to the initial sample data. The original sample data is converted into a number of fuzzy granules, and the data information of each granule sample *G is* characterized by three parameter values (*a*,*m*,*b*). In the second step, the SVM model is applied to regression prediction of the parameters associated with the fuzzy information granules and the parameter values obtained from the regression prediction are used to represent the interval of change in the time series. The flow of the proposed GC-SVM combination prediction model is shown in Figure 5.

Because time series data often have streaming characteristics and the collection time interval is not uniform, the fuzzy granulation algorithm based on time axis has more application prospects. Compared with the algorithm based on combined axes, the fuzzy granulation algorithm based on time axis can process a large number of data more efficiently. Because the time axis has certain regularity, the algorithm can be faster while ensuring the processing accuracy. In addition, the fuzzy granulation algorithm based on time axis has low requirements for data preprocessing.

## 5. Experiments and analysis of results

### 5.1. Description of the experimental data set

In order to verify the effectiveness of the proposed GC-SVM combination prediction model on large sample time series data, two typical medical monitoring cases were selected for experimental analysis. experiment I is fetal weight prediction and experiment II is blood pressure prediction. In obstetrics, accurate prediction of neonatal weight is of great importance. The change in fetal weight is one of the most important indicators of fetal development during pregnancy. Accurate prediction of fetal weight can reduce the risk of labor and improve the quality of the birth of the baby.

The dataset for Experiment I was screened from a sample of 3,000 electronic medical records from the obstetrics department of a hospital between January 1, 2016 and December 31, 2016. The sample was screened for singleton, absence of pregnancy syndrome, and exclusion of malformed fetuses. The age distribution of pregnant women ranged from 22 to 43 years and had undergone an ultrasound examination within 72 h prior to delivery. Some of the experimental data of Experiment I are shown in Table 2.

**TABLE 2:** Selected experimental data of Experiment I.

Gravidity	Parity	Height (cm)	Age	Amniotic fluid index (cm)	Abdominal circumference (cm)	Femur length (cm)	Head circumference (cm)
2	0	160	28	7	33.8	7.3	35.1
2	1	173	34	8	35	7.7	36.9
1	0	163	28	5	343	7.6	34.3
1	0	160	29	12	319	7.1	30.1
1	0	153	32	8	33.7	7.6	34.9
1	0	163	30	7	28.1	5.5	26.1
1	0	165	27	8	32.3	7.1	32.2
1	0	170	29	7	33.7	7.2	33.6
1	0	166	33	7	33.2	6.6	32.9
1	0	164	28	8	30.4	6.6	30.1
1	0	152	30	6	33	7.2	34.9
1	0	158	28	8	32	7.1	32.8
1	0	154	1	10	33.9	7.2	34.1
2	0	162	28	8	33.5	7.3	33.2
2	0	146	30	5	31.5	6.8	31.5
1	0	160	32	11	34.2	73	35.3
1	0	153	30	6	30.4	7.2	33.2
1	0	165	32	8	33.5	7.1	34.8
1	0	160	28	3	32.3	7.1	32.2
1	0	165	31	13	347	75	35.8
1	1	160	32	9	31.7	7.1	31.9
1	1	163	29	7	33.6	7.2	35.3
1	0	166	33	10	33.4	76	35.3
1	0	158	34	6	32.4	6.9	32.4
1	0	158	31	13	32.2	6.8	32.4
1	0	159	30	12	31.7	6.4	30.2
1	0	158	31	8	32.7	7.1	32.9
1	0	165	30	8	32.6	7.5	32.2
1	0	170	30	9	33.4	7.5	34.3
1	0	162	31	5	29.3	5.9	26.8

The Experiment II dataset was drawn from 158 hypertensive patients from the same hospitals. The data time frame ranged from January 1, 2017 to July 31, 2017. Blood pressure data were measured at least twice a day during this period. The blood pressure grading used is shown in Table 3. People who are usually in Grade 1 hypertension are more concerned about their blood pressure. By predicting trends in blood pressure, we can provide an early warning of risk for this group of people.

**TABLE 3:** Blood pressure classification.

Category	Systolic blood pressure (mmHg)	Diastolic blood pressure (mmHg)
Normal	120–129	80–84
Normal high pressure	130–139	85–89
Grade I hypertension	140–159	90–99
Grade II hypertension	160–179	100–109
Grade III hypertension	180	110

### 5.2. Data processing and standardization

In this paper, two algorithms are used to complete the missing values and thus perfect the sample data. The first is the most commonly used mean-completion method (Ridenhour et al., 2017). The reason for using mean-completion is that we assume that all missing values are normal, and with a normal distribution, the probability of the mean value occurring is relatively high, thus reducing the bias of interpolation.


(18) $$x_{i\,j}=\frac{\sum_{i=1}^{m}x_{m\,j}^{{}^{\prime }}}{m}$$

where *x*′ denotes the set of samples that do not contain missing values, *x*_*ij*_ denotes the *j*-th feature of sample *i*, and *m* denotes the number of samples.

The second is the nearest neighbor completion method (Harutyunyan et al., 2019). In this paper, Euclidean distance is used to complete the missing relevant parameter values.


(19) $$d_{i\,k}=\sqrt{\sum_{j=1}^{m}{\left(x_{i\,j}-x_{k\,j}^{{}^{\prime }}\right)}^{2}}$$

where *d*_*ik*_ represents the Euclidean distance between *x*_*i*_ and $x_{k}^{\prime }$.

In order to eliminate the influence of units and data magnitude on the model prediction results, data normalization (also known as normalization) is performed before the parameters are entered into the prediction model.


(20) $$\hat{x}=\frac{x-\mu }{\sigma }$$

Where μ indicates the mean of the current feature parameter values and σ indicates the standard deviation of the current feature.

### 5.3. Experimental environment and parameter settings

**FIGURE 6:**
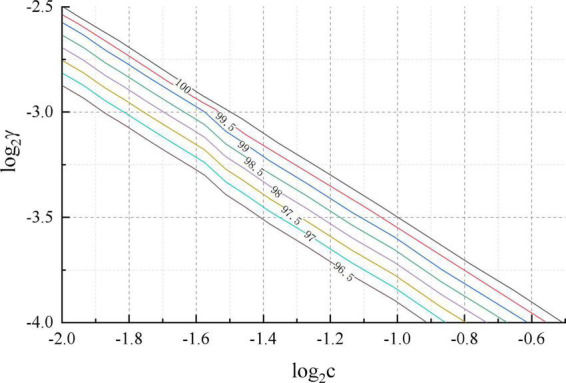
Optimal parameter settings for SVM.

An IBM server with Intel i7 6700k CPU, 8 GB RAM and 300G hard disk was used for this experiment. the system of the IBM server was Ubuntu 14.04 version of Linux operating system. The data cleaning during the experiment was written in Python scripting language, language version 3.5.2. The matlab function used for the cross-validation was crossval. A schematic diagram of the penalty coefficient *c* and the parameter γ for the SVM classification in the prediction model is shown in Figure 6. It can be seen that the optimal penalty coefficients *c* and parameters γ are 0.25 and 0.1758, respectively.

The benchmarking issues in assessing the proposed GC-SVM combination prediction model are mainly related to accuracy and robustness, which are measured using the Mean Absolute Percentage Error (MAPE), Mean Absolute Error (MAE) and Root Mean Square Error (RMSE) as metrics.

### 5.4. Analysis of the results of Experiment I

The proposed GC-SVM combination prediction model was compared with ARMA, SVM and artificial neural network (ANN). The lag of ARMA is 2, the value of AR coefficient is (0.6, −0.5), and the value of MA coefficient is (0.3, −0.4). ANN is a simple three-layer BP neural network model. The number of nodes in the input layer is 3, the number of nodes in the output layer is 1 and the number of nodes in the hidden layer is 1. The performance of all prediction models was averaged over 10 experiments, and the intervals of variation that occurred in the results of the 10 experiments were also recorded. The comparison of the fetal weight prediction models is shown in Table 4. Mean and Euclidean denote the mean-completion and nearest neighbor-completion methods, respectively.

**TABLE 4:** Comparison of fetal weight prediction models.

Predictive models	MAPE (%)	Accuracy (%)
ARMA+Mean	11.18 ± 0.2	52.80 ± 0.1
ARMA+Euclidean	10.96 ± 0.2	53.19 ± 0.1
SVM+Mean	7.69 ± 0.5	62.03 ± 0.5
SVM+Euclidean	7.32 ± 0.3	63.16 ± 0.4
ANN+Mean	6.82 ± 0.3	69.48+0.3
ANN+Euclidean	6.57 ± 0.1	69.37 ± 0.2
GC-SVM+Mean	6.50 ± 0.2	75.90 ± 0.2
GC-SVM+Euclidean	6.22 ± 0.1	77.56 ± 0.1

**FIGURE 7:**
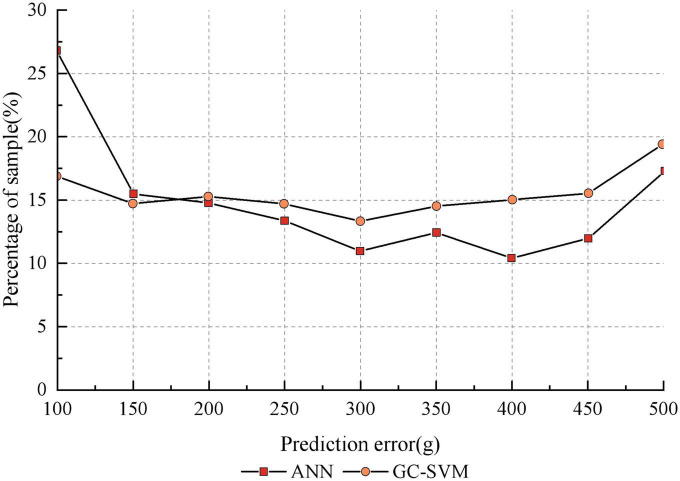
Comparison of robustness between GC-SVM and ANN.

It can be seen that the GC-SVM prediction model reduces the prediction error of the model and improves the stability of the model compared to other prediction models. In order to further compare the robustness of the GC-SVM model with that of the ANN model, the distribution of the two types of models in each error range is analyzed in this paper, as shown in Figure 7. It can be seen that the ANN model is more accurate than the GC-SVM model when the error range is less than 150 g, but the GC-SVM model is more accurate when the error range is greater than 150 g. This indicates that the GC-SVM model can reduce the failure rate of prediction in a larger error range.

### 5.5. Analysis of the results of Experiment II

**FIGURE 8:**
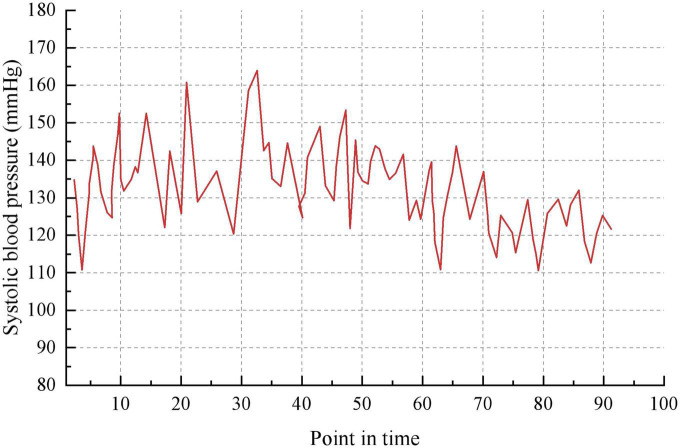
Blood pressure recordings data for 100 consecutive days.

**FIGURE 9:**
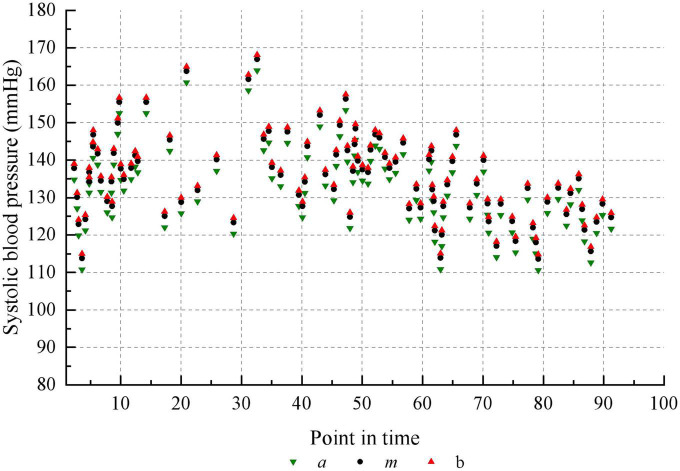
Blood pressure recording data after granulation.

Taking the historical samples of a patient for 100 consecutive days as an example, the original blood pressure time series data are shown in Figure 8. The blood pressure time series data after granulation is shown in Figure 9. To evaluate the performance of various models on the blood pressure time series prediction task, two metrics, RMSE and MAE, were chosen in this paper. Both metrics are calculated such that the smaller the result, the higher the prediction accuracy of the model.

The characteristics of each information grain are characterized by three parameters *a*, *m* and *b*. The parameter *a* represents the minimum value of the change in value over the specified time interval. The parameter *m* represents the average value of the change in value over the specified time interval. The parameter *b* represents the maximum value of the change in value over the specified time interval. It can be seen that the trend in blood pressure after granulation is consistent with the actual trend in blood pressure, indicating that the information has maintained the pattern of the original sample after granulation. The experimental data after granulation can completely characterize the characteristics of the original experimental data, which indicates that the granulation algorithm is feasible. The final prediction results of the four models are shown in Table 5.

**TABLE 5:** Final prediction results for the four models.

Predictive models	Systolic blood pressure (mmHg)		Diastolic blood pressure (mmlg)	
	**MAE (%)**	**RMSE (%)**	**MAE (%)**	**RMSE (%)**
ARIMA+Mean	5.7594	7.7558	3.6197	4.9231
ARMA+Euclidean	5.7284	7.7248	3.5887	4.8921
SVM+Mean	5.6994	7.6958	3.5597	4.8631
SVM+Euclidean	5.6584	7.6548	3.5187	4.8221
ANN+Mean	5.6254	7.6218	3.4857	4.7891
ANN+Euclidean	5.5934	7.5102	3.4283	4.5568
GC-SVM+Mean	5.4276	7.3742	3.4736	4.5267
GC-SVM+Euclidean	5.2534	6.4834	3.2278	4.2832

It can be found that the proposed GC-SVM combination model can make full use of the advantages of both models to capture the trend of blood pressure time series more accurately than the traditional single model. Compared with other prediction models, the combination model has better performance in terms of both prediction precision and stability.

## 6. Conclusion

This paper combines the emerging theory of granular computing with the well-established SVM regression forecasting model and applies it to the forecasting of large sample time series data, in order to uncover potential trends in continuous data. Because of the unique advantages of granular computing in dealing with continuous, complex data, it can compensate for the limitations of traditional SVM in dealing with large sample data. Therefore, this paper proposes to combine granular computing theory with SVM to achieve large sample time series data prediction. First, the initial sample time series is divided into a number of small and continuous time intervals according to the requirements of the actual problem. Each time series is the window concept in the conventional sense of the fuzzy granulation algorithm. The fuzzy granulation algorithm is then used to granulate the data from each window to produce a number of fuzzy information granules. The key to the fuzzy granulation process is the sample data fuzzification process, and this paper employs triangular fuzzy granules. The results of Experiment I of fetal weight prediction show that the GC-SVM combination model can reduce the error rate of prediction in the range of large error. The results of Experiment I of blood pressure time series show that the proposed GC-SVM combination model performs better in prediction accuracy and stability than other prediction models.

## Data availability statement

The raw data supporting the conclusions of this article will be made available by the author, without undue reservation.

## Author contributions

YY conceived and designed the study, collected samples and performed the experiments, analyzed the data, and wrote the manuscript.
